# Etiological spectrum of in-hospital cardiac arrest and its association with clinical outcomes

**DOI:** 10.6026/973206300212841

**Published:** 2025-08-31

**Authors:** Varuna Varma, Rajiv Ratan Singh, Shiv Shanker Tripathi, Utkarsh Kumar Srivastava

**Affiliations:** 1 Department of Cardio Vascular & Thoracic Surgery, Sanjay Gandhi Post Graduate Institute of Medical Sciences, Lucknow, Uttar Pradesh, India; 2 Department of Emergency Medicine, Dr Ram Manohar lohia Institute of Medical Sciences, Lucknow, Uttar Pradesh, India

**Keywords:** In-hospital cardiac arrest, IHCA, cardiac causes, non-cardiac causes, myocardial infarction, arrhythmia, ROSC, survival to discharge, shockable rhythm, CPR, ACLS, neurological outcome, emergency department, hospital resuscitation, cardiac arrest registry

## Abstract

In-hospital cardiac arrest (IHCA) remains a critical medical emergency with persistently poor survival and neurological outcomes despite advances in resuscitation. This prospective study analyzed one year emergency department registry data to classify the leading causes of IHCA and their influence on patient outcomes. Cardiac etiologies, primarily myocardial infarction and arrhythmias, accounted for 55% of cases and were associated with higher ROSC and survival compared to non-cardiac causes. Shockable rhythms markedly improved prognosis, while delayed CPR initiation and arrests in general wards predicted poorer outcomes. Thus, we show the importance of rapid recognition, timely resuscitation and targeted post-arrest care to maximize survival and neurological recovery in IHCA.

## Background:

In-hospital cardiac arrest (IHCA) has become a major cause of morbidity and mortality worldwide despite significant advances in medical technology and resuscitative methods [1]. Cardiac arrest refers to the cessation of effective cardiac mechanical activity leading to loss of circulation and requiring timely intervention [2]. In hospital settings, IHCA presents unique challenges but also opportunities for early detection and intervention due to the presence of trained staff and medical equipment [3]. Each year, hundreds of thousands of patients are affected by IHCA. The American Heart Association (AHA) estimates more than 290,000 adult IHCAs occur annually in the United States alone, yet survival to hospital discharge remains only 20-25% and many survivors suffer severe neurological deficits [4]. IHCA arises from multifactorial etiologies, with both cardiac causes (e.g., myocardial infarction, arrhythmias) and non-cardiac causes (e.g., respiratory failure, sepsis, hemorrhage, metabolic derangements) contributing to outcomes including return of spontaneous circulation (ROSC), survival and neurological recovery [5, 6]. Initial cardiac rhythm is another key determinant of survival. Patients with shockable rhythms such as ventricular fibrillation (VF) and pulseless ventricular tachycardia (VT) have consistently better outcomes compared with those presenting with non-shockable rhythms like asystole and pulseless electrical activity (PEA) [7, 8]. This highlights the need to further understand mechanisms and predisposing factors of IHCA.

Globally, IHCA continues to be one of the most urgent and life-threatening medical emergencies, even in monitored hospital environments equipped with advanced life support systems [9]. Despite decades of improvements and international guidelines-such as the AHA Advanced Cardiovascular Life Support (ACLS) and European Resuscitation Council (ERC) protocols-overall survival has plateaued at 15-25% in high-income countries and remains lower in low-resource settings [10, 11]. Large registries including the Get with the Guidelines-Resuscitation (GWTG-R) in the US and the Swedish Registry of Cardiopulmonary Resuscitation (SRCR) have provided valuable epidemiological data, showing modest survival gains due to improved hospital practice and education. However, the etiological factors influencing outcomes are underrepresented in routine clinical evaluation and management after IHCA [12]. The hospital context of the arrest is also crucial. Arrests in intensive care units (ICUs) or emergency departments (EDs) are associated with better outcomes due to rapid detection and immediate access to resuscitative resources, whereas events in general wards often suffer delays, leading to poorer prognosis [3]. Research gaps remain regarding the direct correlation between etiology and outcomes such as ROSC, survival to discharge and neurological recovery, which are critical for guiding resource allocation, enhancing unit preparedness and tailoring patient-specific resuscitation strategies [4]. Therefore, it is of interest to report the common causes of in-hospital cardiac arrest and their implications on ROSC, survival to discharge and neurological outcomes.

## Material and Methods:

## Study design:

The study was conducted at Dr Ram manohar lohia institute of medical sciences Lucknow, India in the Department of Emergency Medicine a tertiary care hospital with intensive care, emergency and general ward facilities. Ethical approval was taken from institutional ethical committee with IEC number 100/24. Data was collected from medical records and resuscitation logs over a period of one year. This is a Prospective observational study conducted to identify the causes of in-hospital cardiac arrest (IHCA) and assess their impact on patient outcomes. The study involves analysis of clinical data from adult patients who experienced IHCA during a defined period.

## Inclusion criteria:

[1] Patients aged ≥18 years

[2] Patients who experienced cardiac arrest within the hospital setting

[3] Cases where resuscitation was attempted (i.e., activation of a code blue response)

## Exclusion criteria:

[1] Out-of-hospital cardiac arrest (OHCA) cases

[2] Do-not-resuscitate (DNR) orders prior to arrest

[3] Incomplete or missing records

## Data collection:

Data will be extracted from Electronic health records (EHR),Code blue documentation, Hospital cardiac arrest registry and follow the variable records like Demographic details: age, sex, comorbidities, Location of arrest: ICU, ER, general ward, etc., Cause of cardiac arrest (categorized as cardiac or non-cardiac origin), Initial cardiac rhythm (e.g., ventricular fibrillation, asystole, pulseless electrical activity), Time to CPR initiation and defibrillation with the Outcomes of Return of spontaneous circulation (ROSC), Survival to hospital discharge, 30-day survival, Neurological outcome (CPC score).

## Data analysis:

Data will be entered into Microsoft Excel and analyzed using SPSS (Statistical Package for the Social Sciences) version XX.

[1] Descriptive statistics (mean, median, standard deviation) for continuous variables

[2] Frequencies and percentages for categorical variables

[3] Chi-square test and Fisher's exact test for comparison of outcomes based on arrest cause

[4] Logistic regression analysis to determine predictors of survival and neurological recovery. p-value of <0.05 will be considered statistically significant

## Results:

A total of 300 patients who experienced in-hospital cardiac arrest (IHCA) between June 2024 and May 2025 were included in the study (Table 1 - see PDF). Patients with cardiac etiology had significantly higher survival rates (p < 0.01) (Table 2-5 - see PDF). Shockable rhythms (VF/pVT) were significantly associated with ROSC and favorable neurological outcomes (p < 0.001). Longer delay to CPR (>3 min) was associated with poor outcomes (p < 0.05).

## Discussion:

The current paper focuses attention on the fact that the underlying cause of in-hospital cardiac arrest (IHCA) plays a major role in predicting patient outcomes. We found that the most prevalent cause of cardiac arrest was cardiac-related causes, with myocardial ischemia and arrhythmias being the most common and these had better chances of ROSC, survival to hospital discharge and good neurological outcome [6]. Other conditions, including non-cardiac causes such as sepsis, hypoxia and hemorrhage, demonstrated significantly worse results, as indicated in the existing literature. Inferior survival in these groups could be attributed to late detection, rapid decline, or systemic disease. Moreover, patients presenting with shockable rhythms (VF/pVT) had significantly higher survival and neurological prognosis than those with non-shockable rhythms (asystole/PEA), highlighting the role of rapid rhythm identification and defibrillation [8]. In general, these findings motivate the implementation of rapid response systems, early identification of clinical decompensation and specific preventive measures, particularly in high-risk patients with non-cardiac conditions. The aim of this paper was to discuss the pathophysiology of IHCA and its effects on patient outcomes. Our results support the strong impact of etiology, arrest location and initial rhythm on survival and neurological recovery. We showed that 55% of all IHCA cases had a cardiac etiology, of which myocardial ischemia accounted for 30.7% and arrhythmias 14.3%. These patients had significantly higher survival rates compared with the rest (43.6% vs. 19.3%). This concurs with earlier large-scale studies such as the Swedish Registry for Cardiopulmonary Resuscitation (SRCR), which also reported superior survival in cardiac-origin arrests [13]. Cardiac causes are often more predictable and occur in monitored environments such as ICUs or cardiology units, allowing quicker recognition and timely interventions including defibrillation and pharmacologic therapy. In contrast, non-cardiac causes-such as sepsis, hypoxia and hemorrhage-tend to present with systemic deterioration, are frequently associated with non-shockable rhythms, delayed responses and multi-organ dysfunction, leading to lower survival and poorer neurological outcomes [13]. The type of initial cardiac rhythm remains a critical determinant of outcome. In our study, only 25% of patients presented with shockable rhythms (VF/pVT), which were strongly associated with favorable outcomes. This aligns with American Heart Association (AHA) data showing survival rates of up to 50% for VF/VT compared to less than 15% for asystole or PEA [14]. Non-shockable rhythms, which comprised 75% of our cases, are typically linked to non-cardiac etiologies and severe underlying illness. Their poor prognosis is likely due to delayed recognition, absence of electrical activity and the challenge of managing reversible causes [4]. The location of arrest also significantly influenced outcomes. Patients experiencing IHCA in ICUs or emergency departments had better chances due to immediate CPR, advanced resuscitative tools and skilled personnel, whereas arrests in general wards were often associated with delays, resulting in lower ROSC and survival [5]. Furthermore, our data indicated that a delay of more than 3 minutes in initiating CPR was associated with a statistically significant reduction in survival and neurological recovery, underscoring the need for hospital-wide training, early warning systems and rapid response teams. Among patients who survived to discharge, those with cardiac causes had higher rates of favorable neurological recovery (CPC 1-2: 33.9%) compared to those with non-cardiac causes (11.9%). In hospital cardiac arrest is caused mainly by cardiac and pulmonary causes, outcome depends on the cause, with a big variability [15]. The disparity in neurological outcomes reinforces the importance of structured post-resuscitation pathways and standardized prognostication protocols.

## Clinical implications:

The findings of this study have several important implications:

[1] Early identification of at-risk patients, particularly those with non-cardiac illness, can improve monitoring and preemptive interventions.

[2] Hospitals should invest in rapid response systems, staff training and early warning tools to identify patient deterioration.

[3] Greater emphasis is needed on post-arrest care protocols, especially in patients with ROSC, to optimize neurological recovery and reduce long-term disability.

[4] Stratifying patients based on cause and rhythm can aid in personalized resuscitation strategies and prognostication.

## Future directions:

Future studies should include multicenter prospective designs with standardized definitions and protocols. Research into biomarkers, machine learning models and real-time monitoring systems could help in earlier identification of deterioration. Additionally, quality improvement initiatives targeting non-cardiac causes of IHCA may have the greatest potential to improve outcomes.

## Conclusion:

Cardiac-related IHCA, especially with shockable rhythms, leads to better survival and a neurological outcome is shown. Timely CPR and ACLS in monitored settings significantly enhance prognosis. Future protocols should emphasize early detection, staff training and etiology-based resuscitation strategies.
